# Plasma Cathepsin S and Cystatin C Levels and Risk of Abdominal Aortic Aneurysm: A Randomized Population–Based Study

**DOI:** 10.1371/journal.pone.0041813

**Published:** 2012-07-23

**Authors:** Bing-Jie Lv, Jes S. Lindholt, Xiang Cheng, Jing Wang, Guo-Ping Shi

**Affiliations:** 1 Institute of Cardiology, Union Hospital, Tongji Medical College of Huazhong University of Science and Technology, Wuhan, China; 2 Department of Medicine, Brigham and Women's Hospital and Harvard Medical School, Boston, Massachusetts, United States of America; 3 Vascular Research Unit, Department of Vascular Surgery, Viborg Hospital, Viborg, Denmark; University Medical Center Utrecht, Netherlands

## Abstract

**Background:**

Human abdominal aortic aneurysm (AAA) lesions contain high levels of cathepsin S (CatS), but are deficient in its inhibitor, cystatin C. Whether plasma CatS and cystatin C levels are also altered in AAA patients remains unknown.

**Methods and Results:**

Plasma samples were collected from 476 male AAA patients and 200 age–matched male controls to determine CatS and cystatin C levels by ELISA. Student's *t* test demonstrated higher plasma levels of total, active, and pro–CatS in AAA patients than in controls (*P*<0.001). ROC curve analysis confirmed higher plasma total, active, and pro–CatS levels in AAA patients than in controls (*P*<0.001). Logistic regression suggested that plasma total (odds ratio [OR] = 1.332), active (OR = 1.21), and pro–CatS (OR = 1.25) levels were independent AAA risk factors that associated positively with AAA (*P*<0.001). Plasma cystatin C levels associated significantly, but negatively, with AAA (OR = 0.356, *P*<0.001). Univariate correlation demonstrated that plasma total and active CatS levels correlated positively with body–mass index, diastolic blood pressure, and aortic diameter, but negatively with the lowest ankle–brachial index (ABI). Plasma cystatin C levels also correlated negatively with the lowest ABI. Multivariate linear regression showed that plasma total, active, and pro–CatS levels correlated positively with aortic diameter and negatively with the lowest ABI, whereas plasma cystatin C levels correlated negatively with aortic diameter and the lowest ABI, after adjusting for common AAA risk factors.

**Conclusions:**

Correlation of plasma CatS and cystatin C with aortic diameter and the lowest ABI suggest these serological parameters as biomarkers for human peripheral arterial diseases and AAA.

## Introduction

The pathogenesis of abdominal aortic aneurysm (AAA) involves extensive inflammatory cell infiltration, proteolytic enzyme secretion, and arterial wall extracellular matrix (ECM) protein degradation. Elastins and collagens are among the most abundant and best–studied ECM proteins that form the base constituent of the arterial wall. Proteolytic degradation of these ECM proteins leads to aortic expansion and rupture. In human AAA, decreased elastin immunoreactivity and the presence of elastin degradation associate with increased inflammatory–cell infiltration [1]. Serum elastin peptide levels correlate with AAA growth rate [2] and predict late rupture [3]. Type I collagen degradation products are also increased in AAA lesions – particularly those from patients with growing or ruptured AAAs [4]. Serum amino terminal pro–peptide of type III collagen levels are significantly higher in AAA patients than in those without AAA [5], and significantly and positively associated with AAA growth rate (r = 0.55) among patients with asymptomatic small AAA [6], [7]. Blood levels of elastin peptide, collagen peptide, and initial AAA size together may predict the risk of future AAA surgical repair [7].

Cathepsin S (CatS), CatK, and CatL are probably the most potent mammalian elastases; CatK and CatL are also potent collagenases [8]. We first detected increased levels of CatS, CatK, and CatL in human AAA lesions [9], [10]. Other cathepsins, such as CatH and CatB protein levels, are also more than 3–fold higher in the aortic wall in AAA patients than in those from patients with arterial occlusive diseases [11]. In contrast, their endogenous inhibitor, cystatin C, was greatly reduced or deficient in human AAA lesions or in blood [4], [10], [11]. We also showed recently that mice lacking either CatK or CatL were resistant to aortic elastase perfusion–induced experimental AAA [12], [13]. Although not reported, CatS deficiency also protected mice from aortic elastase perfusion–induced or Ang–II perfusion–induced experimental AAA (R.W. Thompson, Y. Qin, and G–P. Shi, unpublished observations). In contrast, in angiotensin II perfusion–induced AAA in apolipoprotein E–null mice, cystatin C deficiency significantly expedited AAA formation [14]. These observations suggest that cysteinyl cathepsins play an important role in human AAA pathogenesis. But whether we can control AAA growth in humans by targeting these cathepsins pharmacologically, or whether their blood concentrations serve as biomarkers for AAA inflammation or lesion progression, remains unknown.

Although computed tomography (CT), angiography, and ultrasound remain conventional methods for detecting human AAA, biomarker screenings have become more common and cost-effective for evaluating the progression of coronary heart disease, diabetes, and other associated complications. AAA shares many risk factors with other cardiovascular diseases and metabolic disorders. Hypertensive men have a high risk of developing AAA [15]. Hypertensive rats developed aortic elastase perfusion–induced AAA much faster than normotensive rats [16]. Cigarette smoking is another common risk factor of AAA and other cardiovascular diseases; smoking history associates strongly with AAA growth in humans (*P* = 0.003) [17]. In mouse elastase perfusion–induced experimental AAA, exposure to cigarette smoking increased aortic elastin degradation and enhanced AAA expansion by 30% [18]. Statins are commonly used as lipid–lowering agents in patients with coronary heart diseases [19]. While simvastatin and atorvastatin reduce aortic wall metalloproteinase (MMP) expression and suppress experimental AAA in mice [20]–[22], pravastatin increases human AAA lesion protein levels of MMP–8, MMP–9, and CatB [23]. The current study was designed to examine whether plasma CatS and cystatin C levels associate with AAA size or growth rate when hypertension, smoking, statin use, and several other common human AAA risk factors were considered, using a randomized cohort from a regional population AAA screening trial.

**Table 1:** Demographic factors and potential confounders as well as aortic diameters, lowest ABI, growth rates, and serological findings are shown and compared between cases with AAA and controls.

	Controls (N = 200)		AAA (N = 476)		
****Dichotomous variables****	****No****	****Yes****	****No****	****Yes****	****Odds ratio **(**95% C.I., *P*-value****)
Familiar disposition	194	6	444	32	2.26 (0.93–5.50, *P* = 0.066)
Current smoker	166	34	273	203	3.63 (2.41–5.48, *P*<0.001)
Diabetes mellitus	172	28	426	50	0.72 (0.44–1.19, *P* = 0.197)
Hypertension	114	86	228	248	1.42 (1.02–1.98, *P* = 0.040)
Use of β–blocker	154	46	339	137	1.34 (0.91–1,99, *P* = 0.133)
Use of ACE inhibitors	158	42	348	128	1.38 (0.93–2.06, *P* = 0.108)
Use of statins	130	70	255	221	2.15 (1.53–3.03, *P*<0.001)
ABI <0.90	200	0	352	121	8.53 (4.08–17.8, *P*<0.001)
**Continuous variables**	**Mean**	**SD**	**Mean**	**SD**	**Students t-test, ** ***P*** **-value**
BMI (kg/m^2^)	26.22	3.29	27.17	3.51	0.001
Systolic BP (mmHg)	148.3	19.53	155,3	21.26	<0.001
Diastolic BP (mmHg)	81.1	10.57	87.8	11.91	<0.001
Lowest ABI	1.10	0.12	0.94	0.19	0.012
Max aortic diameter (mm)	18.3	3.29	40.6	11.7	<0.001
Growth rate (mm/year)			2.37	2.53	
**Serological parameters**	**Mean**	**SD**	**Mean**	**SD**	**Students t-test, ** ***P*** **-value**
Total CatS (ng/mL)	10.7	3.67	14.7	4.25	<0.001
Pro-CatS (ng/mL)	3.05	2.19	3.74	2.00	<0.001
Active CatS (ng/mL)	7.78	3.37	11.0	3.70	<0.001
Cystatin C (ng/mL)	948	497	976	550	0.543
Ln [Cystatin C] (ng/mL)	6.75	0.44	6.77	0.47	0.724

**Figure 1:**
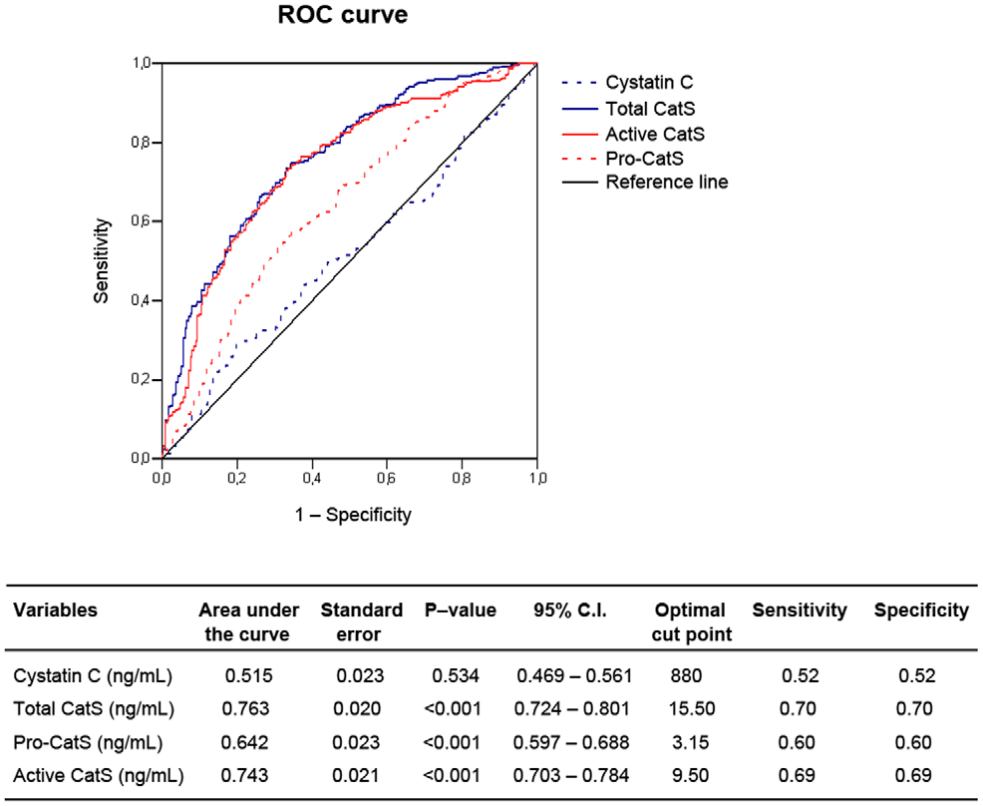
ROC curve analysis for plasma total CatS, pro–CatS, active CatS, and cystatin C levels in predicting AAA. AUC (area under the ROC curve), and optimal sensitivities and specificities of each serological parameter are shown in the associated table.

## Materials and Methods

### Study population

In an ongoing randomized population–based screening trial for AAA, peripheral arterial disease (PAD), and hypertension in more than 50,000 men 65–74 years of age in the mid–region of Denmark [24], baseline plasma samples were taken consecutively at diagnosis of 476 AAA patients and in 200 age–matched controls without AAA or PAD. AAA was defined as having maximal aortic diameter greater than 30 mm, and PAD was defined as an ankle–brachial index (ABI) lower than 0.90. AAA cases among first–degree relatives, smoking status, coexisting diabetes mellitus, hypertension, and use of β–blockers, angiotensin–converting enzyme (ACE) inhibitors, and statins were recorded. Body–mass index (BMI) and systolic and diastolic blood pressure were also measured and recorded. Ankle systolic blood pressure also was measured as previously validated and reported [25], and maximal anterior–posterior diameter of the infrarenal aorta was measured in the peak of the systole from the inner edge to inner edge of aorta. The lowest ABI was calculated as the lowest recorded ankle blood pressure divided by the brachial systolic blood pressure. Patients with AAA less than 50 mm were offered annual control scans by the screening team; patients with AAA measuring 50 mm or larger were referred for a CT–scan and vascular surgical evaluation. The respective departments of vascular surgery organized surveillance for those who did not undergo surgery. The interobserver variation of aortic diameter measurements was 1.52 mm [26]. Growth rates of small AAA in patients kept under surveillance were calculated by individual linear regression analysis, using all observations. Blood samples were centrifuged at 3000 g for 12 minutes, aliquoted, and stored at –80°C until analysis was performed. Written informed consent was obtained from all subjects before participation, and the study was approved by the Local Ethics Committee of the Viborg Hospital, Denmark, and performed in accordance with the Helsinki Declaration. Use of non–coded human samples was also approved by the Partners Human Research Committee, Boston, Massachusetts, USA.

**Table 2:** Logistic regression analysis of total plasma cathepsin S as independent biomarker of aneurysmal disease.

Variables	B	S.E.	*P*-Value	Exp(B)	95,0% C.I. for EXP(B)	
					Lower	Upper
Total CatS (ng/mL)	0.287	0.036	<0.001	1.332	1.242	1.429
Age (year)	0.082	0.041	0.046	1.085	1.002	1.176
Familiar AAA (No = 0, Yes = 1)	0.784	0.570	0.169	2.190	0.716	6.691
Current smoking (No = 0, Yes = 1)	1.450	0.276	<0.001	4.264	2.480	7.331
Diabetes mellitus (No = 0, Yes = 1)	−0.827	0.363	0.023	0.437	0.215	0.891
Hypertension (No = 0, Yes = 1)	0.342	0.254	0.178	1.408	0.855	2.317
ACE-inhibitor (No = 0, Yes = 1)	0.234	0.292	0.423	1.263	0.713	2.238
Beta-blocker (No = 0, Yes = 1)	0.032	0.277	0.907	1.033	0.601	1.776
Use of statins (No = 0, Yes = 1)	0.842	0.251	0.001	2.322	1.420	3.798
Systolic BP (mmHg)	−0.005	0.008	0.486	0.995	0.980	1.010
Diastolic BP (mmHg)	0.069	0.014	<0.001	1.071	1.041	1.101
PAD (No = 0, Yes = 1)	1.837	0.425	<0.001	6.277	2.730	14.431
Body mass index (kg/m^2^)	0.059	0.036	0.099	1.061	0.989	1.138
Ln [Cystatin C] (ng/mL)	−1.034	0.263	<0.001	0.356	0.212	0.595
Constant	−9.293	3.544	0.009	<0.001		

**Figure 2:**
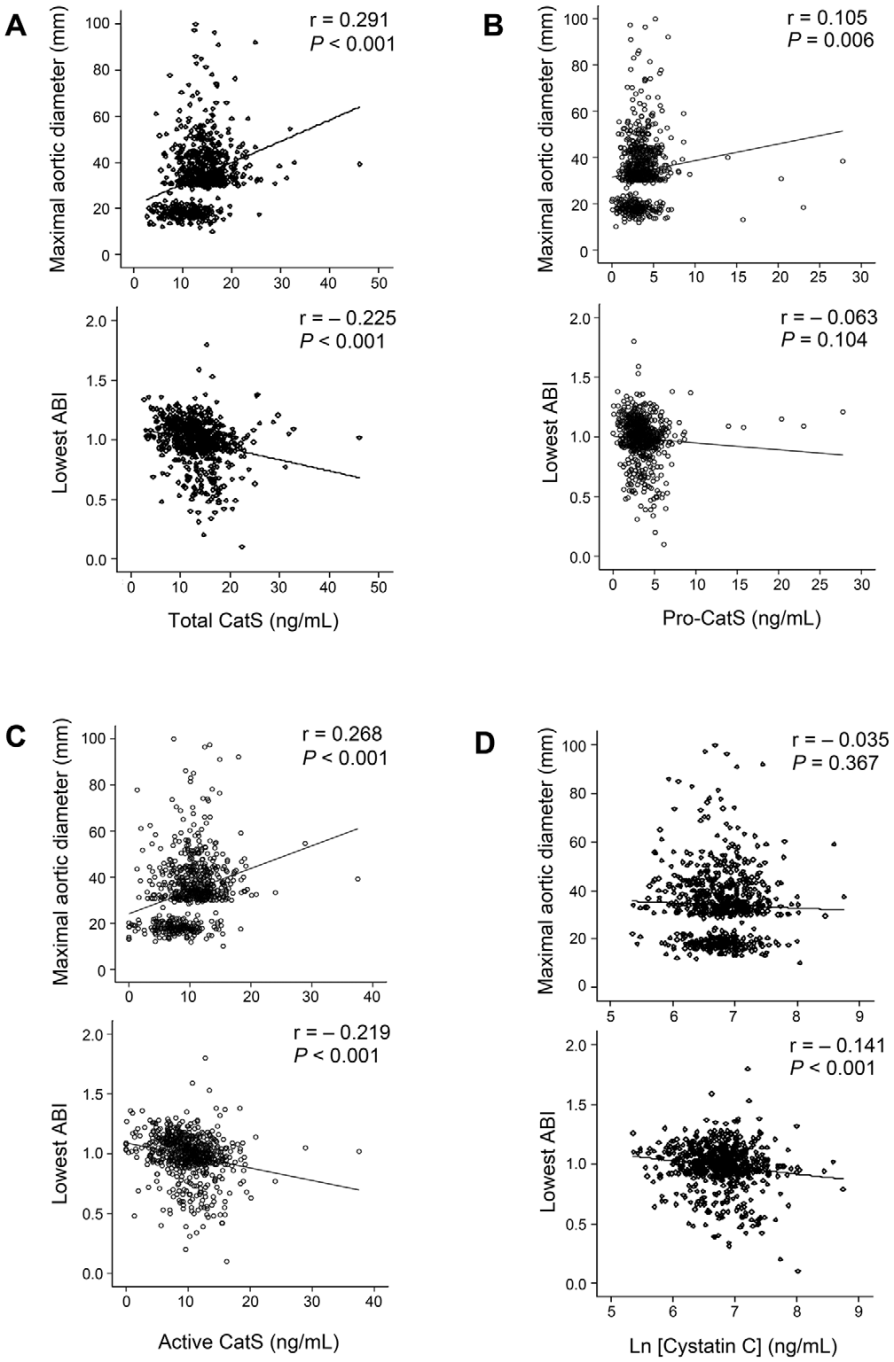
Scatter plots of Pearson correlation analysis of plasma total CatS (**A**)**, pro–CatS** (**B**)**, active CatS** (**C**)**, and cystatin C** (**D**) **levels with maximal aortic diameters and lowest ABI.** Both correlation coefficient and *P* values are indicated for each analysis.

### ELISA

Plasma total CatS, pro–CatS, and cystatin C levels were determined blindly using ELISA DueSet kits from R&D Systems (Minneapolis, MN) (catalog numbers DY1183, DY2227, and DY1196) according to the manufacturer's instructions. Levels of plasma active CatS (also called mature CatS) were determined by subtracting levels of pro–CatS from those of total CatS.

**Table 3:** Univariate correlation analysis of potential serological biomarkers, BMI, blood pressure, maximal aortic diameter, lowest ABI and aneurysmal growth rate.

Serological parameters	Statistical method	BMI	Systolic BP	Diastolic BP	Aorta diameter	Lowest ABI	Growth rate
Ln [Cystatin C] (ng/mL)	Pearson correlation coefficient (r)	−0.018	−0.055	−0.011	−0.035	−0.141	−0.021
	*P*-value	0.635	0.161	0.773	0.367	<0.001	0.707
Total CatS (ng/mL)	Pearson correlation coefficient (r)	0.101	0.030	0.100	0.291	−0.225	−0.026
	*P*-value	0.009	0.438	0.010	<0.001	<0.001	0.633
Pro-CatS (ng/mL)	Pearson correlation coefficient (r)	0.054	−0.046	−0.036	0.105	−0.063	−0.020
	*P*-value	0.166	0.233	0.361	0.006	0.104	0.971
Active CatS (ng/mL)	Pearson correlation coefficient (r)	0.084	0.047	0.115	0.268	−0.219	−0.032
	*P*-value	0.030	0.228	0.003	<0.001	<0.001	0.541

### Statistics

Dichotomous variables were expressed as proportions and compared by the chi–square test, and reported as odds ratios. Probability plots (not shown) were used to determine whether continuous variables were normally distributed, and compared between controls and cases by Student's *t*–test. Receiver–operator characteristic (ROC) curve analyses were performed non–parametrically to test the predictive value of the tests, concerning the prediction of AAA cases. For analyses of the ROC curves, the null hypothesis was that the test performed similarly to the diagonal line – i.e., the area under the curve was 0.5. If the lowest 95% confidence limit for the area under the curve was above 0.5, a significant predictive test was present. The optimal cut–off points were determined, and the respective sensitivity and specificity were calculated. The potential markers were then tested as independent predictors of AAA by logistic regression analysis, adjusting for AAA among first–degree relatives; smoking status; coexisting diabetes mellitus; hypertension; use of β–blockers, ACE inhibitors, or statins; BMI; systolic blood and diastolic blood pressure; and the lowest recorded ABI. The associations between the potential serological biomarkers were then correlated to maximal aortic diameter, lowest ABI, and AAA growth rate by Pearson's correlation analysis. The best potential serological biomarker was then tested for independent association with maximal aortic diameter, lowest ABI, and AAA growth rate, respectively, by multivariate linear regression analyses, adjusting for the aforementioned potential AAA confounders.

**Table 4:** Multivariate linear regressions analyses of aortic diameter, lowest ABI and growth rate as dependent variables.

Dependent variable: Aortic diameter	Unstandardized coefficients		Standardized coefficients (Beta)	*P*-Value
	B	Std. Error		
(Constant)	10.115	9.294		0.277
Total CatS (ng/mL)	0.782	0.117	0.256	<0.001
Familiar AAA (No = 0, Yes = 1)	1.831	2.223	0.030	0.410
Current smoking (No = 0, Yes = 1)	3.593	1.090	0.124	0.001
Diabetes mellitus (No = 0, Yes = 1)	−3.616	1.636	−0.084	0.027
Hypertension (No = 0, Yes = 1)	0.437	1.132	0.016	0.700
Use of ACE inhibitor (No = 0, Yes = 1)	2.271	1.256	0.071	0.071
Use of β-blocker (No = 0, Yes = 1)	0.903	1.213	0.029	0.457
Use of statins (No = 0, Yes = 1)	3.607	1.106	0.130	0.001
Systolic blood pressure (mmHg)	−0.061	0.032	−0.092	0.059
Diastolic blood pressure (mmHg)	0.344	0.057	0.297	<0.001
Peripheral arterial disease (No = 0, Yes = 1)	1.640	1.314	0.047	0.212
Body mass index (kg/m^2^)	0.440	0.152	0.111	0.004
Ln [Cystatin C] (ng/mL)	−3.412	1.123	−0.115	0.002

Dependent variable: Lowest ABI	Unstandardized coefficients		Standardized coefficients (Beta)	*P*-Value
	B	Std. Error		
(Constant)	1.350	0.127		<0.001
Total CatS (ng/mL)	−0.005	0.002	−0.124	0.002
Familiar AAA (No = 0, Yes = 1)	−0.021	0.030	−0.026	0.485
Current smoking (No = 0, Yes = 1)	−0.092	0.015	−0.237	<0.001
Diabetes mellitus (No = 0, Yes = 1)	0.009	0.022	0.016	0.683
Hypertension (No = 0, Yes = 1)	−0.033	0.015	−0.090	0.031
Use of ACE inhibitor (No = 0, Yes = 1)	−0.010	0.017	−0.024	0.548
Use of β-blocker (No = 0, Yes = 1)	−0.024	0.017	−0.057	0.144
Use of statins (No = 0, Yes = 1)	−0.036	0.015	−0.098	0.016
Systolic blood pressure (mmHg)	−0.002	0.000	−0.173	<0.001
Diastolic blood pressure (mmHg)	0.002	0.001	0.113	0.029
Body mass index (kg/m^2^)	0.005	0.002	0.097	0.013
Aortic diameter (mm)	−0.002	0.001	−0.170	<0.001
Ln [Cystatin C] (ng/mL)	−0.029	0.015	−0.073	0.046

Dependent variable: Aneurysmal growth rate	Unstandardized coefficients		Standardized coefficients (Beta)	*P*-Value
	B	Std. Error		
(Constant)	3.316	2.674		0.216
Total CatS (ng/mL)	0.007	0.032	0.013	0.829
Familiar AAA (No = 0, Yes = 1)	0.241	0.564	0.025	0.669
Current smoking (No = 0, Yes = 1)	0.887	0.292	0.183	0.003
Diabetes mellitus (No = 0, Yes = 1)	−0.563	0.516	−0.065	0.276
Hypertension (No = 0, Yes = 1)	0.175	0.313	0.036	0.577
Use of ACE inhibitor (No = 0, Yes = 1)	−0.293	0.359	−0.052	0.415
Use of β-blocker (No = 0, Yes = 1)	−0.326	0.335	−0.060	0.332
Use of statins (No = 0, Yes = 1)	0.216	0.303	0.045	0.476
Systolic blood pressure (mmHg)	−0.006	0.009	−0.055	0.476
Diastolic blood pressure (mmHg)	0.029	0.016	0.142	0.071
Body mass index (kg/m^2^)	−0.040	0.045	−0.055	0.375
Peripheral arterial disease (No = 0, Yes = 1)	−0.309	0.323	−0.057	0.339
Ln [Cystatin C] (ng/mL)	−0.273	0.323	−0.051	0.399

## Results

### Increased plasma CatS levels in AAA patients

Of 50,000 volunteers, 25,000 were randomized for screening for PAD, AAA, and hypertension – among which, approximately 75% attended the screening [24]. Of the first 476 consecutively diagnosed cases of AAA, 385 had small AAA (aortic diameters smaller than 50 mm) and were offered surveillance ranging from 0.52 to 3.1 years, with an average of 1.69±0.57 (mean ± SD) years. Patients with AAA measuring 50 mm or more were referred for a CT scan and to the vascular surgical department to be evaluated for potential repair. Those not given a surgical referral were further followed by the department. Such growth data were not included in this study. Demographic factors and potential confounders, as well as aortic diameters, lowest ABI, growth rates, and serological findings are shown and compared between cases and controls in Table 1. The mean ages were 70.0±2.8 (mean ± SD) years and 69.6±2.8 (mean ± SD) years among those without and with AAA, respectively. Plasma cystatin C levels needed logarithmic transformation to become normally distributed.

In the study population, there were significantly more smokers (odds ratio [OR]: 3.63 [2.41–5.48, 95% C.I.], *P*<0.001) and hypertensives (OR: 1.42, [1.02–1.98, 95% C.I.], *P* = 0.04) among AAA patients than among controls. There were also more users of β–blockers, ACE inhibitors, and statins among AAA patients than among controls. Statin users were significantly more frequent among AAA patients than among controls (OR: 2.15, [1.53–3.03, 95% C.I.], *P*<0.001). AAA patients also had significantly higher values of BMI (*P* = 0.001) and systolic (*P*<0.001) and diastolic (*P*<0.001) blood pressures, but their lowest ABI (OR: 8.53, [4.08–17.80, 95% C.I.], *P*<0.001) values were significantly lower compared with controls (Table 1). As we anticipated, plasma total CatS (*P*<0.001), pro–CatS (*P*<0.001), and active CatS (*P*<0.001) levels were significantly higher in patients with AAA than in controls. We did not see significant differences in plasma cystatin C levels, however, between the two groups – either in direct measurement or after logarithmic transformation (Table 1).

### High plasma CatS levels predict AAA and are independent AAA risk factors

ROC curve analysis demonstrated the significant differences in human plasma total CatS, pro–CatS, and active CatS levels between AAA patients and controls (AUC [area under the ROC curve] = 0.76, *P*<0.001, AUC = 0.64, *P*<0.001, and AUC = 0.74, *P*<0.001, respectively), with optimal sensitivities and specificities of 0.70 and 0.70 *versus* 0.60 and 0.60 *versus* 0.69 and 0.69, respectively, but not plasma cystatin C level (AUC = 0.52, *P* = 0.534) (Figure 1). Logistic regression analysis showed that plasma total CatS, pro–CatS, and active CatS levels are independent risk factors for aneurysmal disease, and associated significantly and positively with AAA with OR of 1.33 (95% C.I.: 1.24–1.43, *P*<0.001); 1.25 (95% C.I.: 1.10–1.41, *P*<0.001); and 1.21 (95% C.I.: 1.14–1.30, *P*<0.001), respectively (only total CatS is shown in Table 2). Among other tested variables, age, current smoking, statin use, diastolic blood pressure, and PAD all associated positively with the risk of AAA. PAD and smoking were the most potent risk factors for AAA, with OR 6.277 (95% C.I.: 2.730–14.431, *P*<0.001) and 4.264 (95% C.I.: 2.480–7.331, *P*<0.001), respectively. In contrast, diabetes status and logarithmized cystatin C levels associated significantly but negatively with AAA, with OR 0.437 (95% C.I.: 0.215–0.891, *P* = 0.023) and 0.356 (95% C.I.: 0.212–0.595, *P*<0.001) for diabetes and logarithmized plasma cystatin C levels, respectively (Table 2).

### Correlations of plasma CatS and cystatin C concentrations with AAA diameter and lowest ABI

Univariate correlation analysis of the potential serological biomarkers (CatS and cystatin C), BMI, blood pressure, maximal aortic diameter, lowest ABI, and aneurysmal growth rate demonstrated that both plasma total CatS and active CatS levels correlated significantly and positively with BMI, diastolic blood pressure, and aortic diameter, and negatively with lowest ABI. Pro–CatS levels correlated significantly and positively only with aortic diameter. None of these plasma CatS values associated with AAA growth rate or systolic blood pressure (Table 3). In contrast, plasma cystatin C levels correlated significantly and negatively with lowest ABI, but not with the other tested variables (Table 3). Scatter plots showed significant positive associations of plasma total CatS (r = 0.291, *P*<0.001), active CatS (r = 0.268, *P*<0.001), and pro–CatS levels (r = 0.105, *P* = 0.006) with maximal aortic diameters and negative associations of total CatS (r = −0.225, *P*<0.001) and active CatS (r = −0.219, *P*<0.001) levels with lowest ABI values (Figure 2A–2C). In contrast, plasma cystatin C levels correlated significantly and negatively with lowest ABI (r = −0.141, *P*<0.001), but not with maximal aortic diameters (r = −0.035, *P* = 0.367) (Figure 2D). After depleting 200 age-matched controls from this population, which may reduce the power of association analysis and increase the risk of a type 2 error, only plasma cystatin C remained significantly and negatively associated with lowest ABI (r = −0.160, *P*<0.001). All other associations lost their statistical significance (data not shown).

We used multivariate linear regression analysis to determine whether plasma CatS or cystatin C levels associated with AAA size, lowest ABI, or aneurysmal growth rate when these vascular parameters were considered as individual dependent variables. When aortic diameter alone was considered as a dependent variable, we found that levels of plasma total CatS (*P*<0.001), pro–CatS (*P* = 0.001), and active CatS (*P*<0.001) correlated significantly and positively with aortic diameter after adjusting for all other variables, including familial AAA; current smoking; diabetes mellitus; hypertension; use of ACE inhibitors, β–blockers, or statins; systolic and diastolic blood pressures; PAD; BMI; and plasma cystatin C levels (only total CatS is shown in Table 4). In contrast to univariate correlation analysis, which showed no significant association between plasma cystatin C and aortic diameters (*P* = 0.367, Table 3), a multivariate linear regression test showed that plasma cystatin C levels correlated significantly and negatively with aortic diameter after the same adjustment of all remaining variables (*P* = 0.002, Table 4). Plasma cystatin C levels therefore may be confounded heavily by many common AAA risk factors.

Among the list, smoking, statin use, diastolic blood pressure, and BMI associated positively and significantly with aortic diameter, whereas coexisting diabetes mellitus associated negatively with aortic diameter, as did cystatin C (Table 4). When lowest ABI was used as a dependent variable, plasma total CatS (*P* = 0.002), active CatS (P = 0.001), and cystatin C (*P* = 0.046) associated significantly and negatively with lowest ABI after adjustments for familial AAA; smoking; coexisting diabetes mellitus; hypertension; use of ACE inhibitors, β–blockers, or statins; systolic and diastolic blood pressures; BMI; and aortic diameter. We did not observe a significant association concerning pro–CatS.

Among all other tested variables, smoking (*P*<0.001), hypertension (*P* = 0.031), statin use (*P* = 0.016), systolic blood pressure (*P*<0.001), and aortic diameter (*P*<0.001) associated significantly and negatively with lowest ABI, while diastolic blood pressure and BMI associated significantly and positively with lowest ABI after adjustment for all other variables in Table 4. When aneurysmal growth rate was considered as a dependent variable, however, only current smoking associated significantly and positively with aneurysmal growth rate (*P* = 0.003). None of the other variables reached statistical significance (Table 4). Smoking appears to be a significant confounding factor of AAA. To assess whether higher plasma CatS levels in AAA patients (*P*<0.001, Table 1) were due to there being more smokers among AAA patients (*P*<0.001, Table 1) than among controls, we excluded all smokers from AAA and control populations. Among remaining non-smokers, 274 AAA patients still had significantly higher plasma total CatS levels than those in 167 remaining controls (14.56±4.20 ng/mL *vs*. 10 56±3.69 ng/mL, mean ± SD, *P*<0.001).

## Discussion

CatS was one of the first cysteinyl cathepsins discovered in human AAA lesions [10]. Its activities in degrading elastin [27], fibronectin [28], collagen [29], and laminin [29] suggest strongly its involvement in AAA formation and progression. While the expression of MMPs and their tissue inhibitors, TIMP–1 and TIMP–2, increase synchronously in human AAA lesions [30], [31], expression of CatS and its endogenous inhibitor cystatin C is regulated oppositely in human AAA lesions. Several studies demonstrated increased CatS expression but greatly decreased cystatin C expression, or its deficiency, in human AAA lesions [4], [10], [11]. The current study analyzed two forms of CatS in human plasma samples and demonstrated significantly elevated levels of pro–CatS and its activated form (Table 1). While pro–CatS seems a weaker biomarker of human AAA, with optimal sensitivity and specificity at 0.60 and 0.60, active CatS and total CatS are much stronger AAA biomarkers with optimal sensitivities and specificities at 0.1 higher than those of pro–CatS (Figure 1). Although no other plasma cysteinyl cathepsins in AAA patients have been reported, increased CatS levels may associate with increased AAA lesion CatS expression [4], [10], [11] and may serve as an independent risk factor and biomarker for human AAA. Indeed, the OR for total CatS, active CatS, and pro–CatS in a logistic regression model (Table 2) correlated significantly with AAA, thereby serving as independent AAA risk factors. Determining the detailed molecular mechanisms by which CatS participates in AAA formation and progression, however, will require more robust analysis in experimental AAA and human AAA lesions.

This study showed that plasma CatS levels (total CatS, pro–CatS, and active CatS) correlated positively with aortic diameters, but negatively with lowest ABI, after adjustment of all potential AAA confounders (Table 4). These observations are consistent with our original hypothesis that more advanced AAA may contain higher levels of CatS in AAA lesions, as well as in the circulation. CatS uses its activities in degrading ECM [27]–[29] and in processing and presenting antigens [32] to promote arterial wall elastinolysis and leukocyte recruitment [33] and angiogenesis [27], and to activate lymphocytes [32] – all which are important in AAA pathogenesis. We have previously shown that patients with atherosclerotic stenosis had significantly higher serum levels of CatS than did patients without cardiovascular complications, before (*P*<0.04) and after adjustments for cystatin C levels, renal functions, smoking, and serum glucose levels (*P* = 0.008) [34]. Consistent with our prior findings, plasma CatS levels (total CatS, pro–CatS, and active CatS) correlated negatively with the lowest ABI in the multivariate linear regression model (β = −0.124, *P* = 0.002) (Table 4). Surprisingly, plasma CatS levels showed no correlation with AAA annual expansion rate in either the univariate correlation analysis (Table 3) or the multivariate linear regression model (Table 4). Although we currently do not have any explanation for this finding at the molecular level, it may result from this study's relatively short observation time of small AAA (1.69±0.57 years, mean ± SD) under surveillance, which could easily have caused failure to demonstrate associations with aneurysmal growth rate. Extended monitoring in the future may increase the power of association analysis between plasma CatS and AAA annual growth rate. But plasma CatS may not associate with AAA growth rate, regardless of the length of surveillance. CatS is a lysosomal protease that degrades ECM *in situ* in the arterial wall. Enlarged AAA lesions may contain increased cell infiltration and proliferation. High AAA growth rate reflects fast lesion cell accumulation and proliferation, which may not affect overall CatS expression. All of these possibilities or study limitations warrant further investigation in this or similar patient populations.

The current study also reflects reduced cystatin C in human AAA lesions [4], [10], [11]. Although we did not see significant differences in plasma cystatin C levels between AAA patients and control patients (Table 1), and plasma cystatin C did not predict AAA (Figure 1), logistic regression analysis did demonstrate a significant correlation of plasma cystatin C with AAA, and cystatin C served as an independent AAA risk factor (OR  = 0.356, *P*<0.001) (Table 2). While univariate correlation showed that cystatin C correlated with lowest ABI (*P*<0.001), but not with AAA size (*P* = 0.367) (Table 3), multivariate linear regression analysis demonstrated a significant and negative correlation with AAA size (β = −0.115, *P* = 0.002). In a relatively smaller randomized mass screening trial (n = 142), Lindholt et al. also found negative correlations between plasma cystatin C levels and human AAA size (r = −0.22) and AAA annual expansion rate (r = −0.24), but no prediction of cases requiring later surgical repair – i.e., AAA larger than 50 mm [35]. Although the current study also showed that cystatin C did not discriminate AAA from ROC curve analysis (sensitivity and specificity are both 0.52), we did not see an association between cystatin C and AAA annual growth rate (*P* = 0.399), likely because of the relatively short AAA observation time discussed above. Nevertheless, this study has strength in its population–based, relatively large sample sizes that make selection bias unlikely and information bias concerning aortic size and ABI minimal, as it used standardized and validated methods for data analysis.
